# Widowhood and mortality: a Danish nationwide register-based cohort study

**DOI:** 10.1017/S2045796020000591

**Published:** 2020-08-03

**Authors:** C. Blanner, A. Mejldal, A. M. Prina, P. Munk-Jørgensen, A. K. Ersbøll, K. Andersen

**Affiliations:** 1 Department of Mental Health, Odense – University Clinic, Mental Health Services Region of Southern Denmark, Odense, Denmark; 2 Institute of Clinical Research, University of Southern Denmark, Odense, Denmark; 3 Department of Health Service and Population Research, Institute of Psychiatry, Psychology & Neuroscience at King's College London, Social Epidemiology Research Group, London, UK; 4 National Institute of Public Health, University of Southern Denmark, Copenhagen, Denmark

**Keywords:** Bereavement, elderly, geriatric psychiatry, survival analysis

## Abstract

**Aims:**

Widowed people have increased mortality compared to married people of the same age. Although most widowed people are of older age, few studies include the oldest old. As life expectancy is increasing, knowledge of widowhood into older age is needed. This study aimed to examine mortality and widowhood in older age by comparing mortality in widowed and married people by sex, age, time since spousal loss and cause of death.

**Methods:**

A Danish register-based matched cohort study of 10% of widowed persons ⩾65 years in the years 2000–2009. For each randomly drawn widowed person, five married persons were matched on sex and age. Mortality rate ratios (MRR) were calculated using Poisson regression, and stratified according to sex and 5-year age intervals. MRRs were furthermore calculated by time since spousal loss and by specific cause of death.

**Results:**

The study included 82 130 persons contributing with 642 914.8 person-years. The overall MRR between widowed and married persons with up to 16 years of follow-up was 1.25 (95% CI 1.23–1.28). At age ⩾95 years for men, and ⩾90 years for women, no differences in mortality rates were seen between widowed and married persons. Mortality in widowed persons was increased for most specific causes of death, with the highest MRR from external causes (MRR 1.53 [1.35–1.74]) and endocrine diseases (MRR 1.51 [1.34–1.70]).

**Conclusions:**

Widowhood was associated with increased mortality in older age for both men and women until age ⩾95 and ⩾90 years, respectively. Increased mortality was observed for almost all causes of death.

## Introduction

Widowed people have increased mortality compared to married people (Moon *et al*., 2011; Shor *et al*., 2012), a phenomenon known as ‘the widowhood effect’ (Sullivan and Fenelon, 2014; Vable *et al*., 2015; Ennis and Majid, 2019). For people at age 65 years or above, the relative increase in mortality in widowed people compared to married people has been estimated to be 11% (Manzoli, 2007).

The risk of mortality seems to be highest immediately after spousal loss (Moon *et al*., 2011) but studies have found ‘a widowhood effect’ even after 25 years of follow-up (Shor *et al*., 2012). Results from different studies are however conflicting, and further knowledge of the longitudinal association is needed (Ennis and Majid, 2019).

Various conditions have been shown to be increased in widowed people compared to married people, including cardiovascular risk factors, chronic pain, cancers, lower functional status and mental disorders (Ennis and Majid, 2019). Also mortality in widowhood has been shown to be increased from various conditions including both acute events such as infections and acute myocardial infarction, and chronic diseases (Elwert and Christakis, 2008). This may be due to reduced social and emotional support, leading to reduced self-care of diseases (Elwert and Christakis, 2008). Furthermore, increased death from external causes in widowed people has been shown (Brenn and Ytterstad, 2016). This is partly due to suicides (Ajdacic-Gross *et al*., 2008) but an increased risk of accidents such as falls has also been found (Elwert and Christakis, 2008; Einio and Martikainen, 2019). Similar to the acute and chronic diseases described above, death from accidents could be related to less self-care and neglect of own needs, as well as be due to increased risk behaviours (Einio and Martikainen, 2019). Reduced self-care, increased risk behaviour and an increased risk of suicide have been associated with depression (Hawton and van Heeringen, 2009; Einio and Martikainen, 2019). As the prevalence of depression is high in widowed people (Kristiansen *et al*., 2019*a*, 2019*b*), this could be a possible explanation for some of the excess mortality in widowhood, and depression could thus be a relevant target for preventive interventions in the future. However, to be able to design and aim relevant interventions, more knowledge of causes of death, also in the oldest old, is needed, in order to understand the pathways into the increased mortality in widowhood.

Few studies of the widowhood effect examine how this is associated with age in the oldest old (Shor *et al*., 2012). In the meta-analysis by Moon *et al*., age was dichotomised as younger than 65 years and 65 years or above (Moon *et al*., 2011). Shor *et al*. studied age also in the older population, more specifically by using 10-year age strata, however, with their oldest age stratum pooling those aged 80 years and above (Shor *et al*., 2012). With the increasing proportion of older people in most societies (United Nations, 2017), and with most widowed people being of older age (The Loomba Foundation, 2015), more knowledge about this group is needed. This is particularly important, as the increased mortality in widowed people has been shown to be increasing through the past decades (Moon *et al*., 2011; Shor *et al*., 2012).

The aim of this study was to examine widowhood and mortality in older age, including the oldest old. The study aimed to (1) examine widowhood and overall mortality compared to married persons by age, sex and time since spousal loss and (2) compare cause-specific mortality between widowed and married persons.

## Methods

This is a Danish register-based matched cohort study. In Denmark, each citizen is assigned a unique lifelong personal identification number (the CPR number) which enables linkage between different registers at an individual level (Pedersen, 2006). Data in this study were drawn from the Danish Civil Registration System (Pedersen, 2006), the Income Statistics Register (Baadsgaard and Quitzau, 2011), the Danish Register of Causes of Death (Helweg-Larsen, 2011) and the Danish National Patient Register (Schmidt *et al*., 2015). For project protocol see Appendix 1.

### Study population

The cohort consisted of a random sample of 10% of people in Denmark who became widowed at age 65 years or above in the period 2000–2009, both years included. Using incidence density sampling, five married persons were matched to each widowed person on age and sex.

Each person was followed in the registers from the date of inclusion until the date of death or censoring, which could be any day after inclusion. Thus, people could be followed for one day up to 16 years. Censoring of widowed persons was due to remarriage, emigration or end of study (31 December 2015). Censoring of married persons was due to becoming widowed, divorced, emigration or end of study. Married persons who became widowed were censored as married and were subsequently re-included as widowed on the date they lost their spouse and were followed as widowed until death or censoring. Each person contributed with person-years (PY) according to their current marital status at all times.

### Exposure

The exposure studied was widowhood. This information was obtained from the Danish Civil Registration System (Pedersen, 2006). A person could be included either as widowed (exposed) or married (unexposed). Exposure status could change during the study from unexposed (married) to exposed (widowed) but not reverse. This strategy was chosen as widowed persons are potentially at an increased risk of death due to the experience of being widowed. Therefore, persons who have been widowed have been exposed to the potential long-term consequences of widowhood and could therefore not be re-included as unexposed.

### Outcome

The outcome was death. Date and cause of death were extracted from the Danish Civil Registration System (Pedersen, 2006) and the Danish Register of Causes of Death (Helweg-Larsen, 2011). The cause of death used was the underlying cause of death, defined as the disease or condition, which started the process that led to death (Helweg-Larsen, 2011). The causes of death were grouped according to ICD-10 categories (World Health Organization, 1992) as follows: (1) Infectious and parasitic diseases [A00–B99], (2) Neoplasms [C00–D48], (3) Diseases of the blood and blood-forming organs and immunological disorders [D50–D89], (4) Endocrine, nutritional and metabolic diseases [E00–E90], (5) Mental and behavioural disorders [F00–F99] and Diseases of the nervous system and sense organs [G00–H95], (6) Diseases of the circulatory system [I00–I99], (7) Diseases of the respiratory system [J00–J99], (8) Diseases of the digestive system [K00–K93], (9) Diseases of the genitourinary system [N00–N99], (10) Injuries and other external causes of morbidity and mortality [S00–Y98], (11) Symptoms signs and ill-defined causes [R00–R99] and (12) Other causes of death [L00–L99, M00–M99]. The ICD-10 chapters of mental and behavioural disorders (F00–F99) and diseases of the nervous system and sense organs (G00–H59) were combined because the vast majority of deaths from these chapters were related to dementia.

### Covariates

Time since spousal loss was examined as different periods of follow-up times from inclusion: 1 week, 1 month, 3 months, 6 months, 1 year, 2 years, 5 years, 10 years and 15 years, respectively. Widowed persons were followed from the date of spousal death (which equals the date of inclusion). Married persons were followed from the date of inclusion (which equals the day they were matched with the widowed person, and thus the date the widowed person lost his/her spouse).

Age was examined in 5-year intervals from 65–69 to ⩾100 years in the analyses stratified according to age. Socioeconomic status (SES) was measured as the total disposable personal income in the year prior to inclusion divided into deciles. Deciles were calculated across the study population in the calendar year of inclusion to enable comparison over the study period. The income from the year prior to inclusion was used to avoid false high estimates in the first year of widowhood due to death grants from pension funds, inheritances, etc. Comorbidity was defined according to the Charlson Comorbidity Index (Charlson, 1987; Charlson *et al*., 1994), applying comorbidity groups in terms of ICD-10 codes in accordance with previous studies using the Charlson Comorbidity Index (Brink *et al*., 2017). For each person, a Charlson Comorbidity Score (CCS) was calculated including diagnoses given up to 5 years prior to inclusion based on data from the Danish National Patient Register (Schmidt *et al*., 2015). For persons who changed exposure status from married to widowed, income and CCS were updated at the time of re-inclusion as widowed.

### Statistical analysis

Mortality rates (MRs) were calculated as the number of deaths/10 000 PY for widowed and married persons, stratified according to sex and 5-year age intervals. Time since spousal loss and specific causes of death were considered. Overall and cause-specific mortality rate ratios (MRR) with 95% confidence intervals (CIs) were calculated using Poisson regression of the number of deaths with logarithmic transformation of PY as offset and adjusted for the potential confounders: age in years, sex, CCS (in categories 0, 1, 2 or ⩾3), income decile and calendar year. In the analyses of cause-specific mortality, a person was censored in case of death by other causes than the cause of death examined, and persons with missing causes of death were excluded. Analyses were conducted using the statistical software package, Stata 15 (2017).

## Results

### Characteristics of the study population

A total number of 82 130 persons were included in the study. Of the total study population, 66.4% were women (*n* = 54 534). The mean age of the study population was 75.8 years (s.d. 6.9) at the time of matching. Further characteristics at the time of matching including income deciles and comorbidity scores are seen in Table 1.

**Table 1.** Baseline characteristics of the study population

	Widowed		Married	
	Male	Female	Male	Female
*n* (% by exposure status)	4476 (34.5)	8488 (65.5)	23 120 (33.4)	46 046 (66.6)
Age (mean (s.d.))	77.0 (7.3)	74.9 (6.5)	77.1 (7.2)	75.2 (6.6)
Income decile distribution^a^ (*n* (%))				
1st Decile	100 (2.2)	1212 (14.3)	664 (2.9)	7602 (16.5)
2nd Decile	52 (1.2)	1043 (12.3)	438 (1.9)	5981 (13.0)
3rd Decile	113 (2.5)	1209 (14.2)	811 (3.5)	6334 (13.8)
4th Decile	335 (7.5)	1075 (12.7)	1762 (7.6)	5671 (12.3)
5th Decile	546 (12.2)	846 (10.0)	2851 (12.3)	4497 (9.8)
6th Decile	646 (14.4)	738 (8.7)	2987 (12.9)	3695 (8.0)
7th Decile	647 (14.5)	654 (7.7)	2671 (11.6)	3382 (7.3)
8th Decile	480 (10.7)	778 (9.2)	2237 (9.7)	3681 (8.0)
9th Decile	718 (16.0)	604 (7.1)	3445 (14.9)	3148 (6.8)
10th Decile	839 (18.7)	329 (3.9)	5254 (22.7)	2055 (4.5)
Charlson Comorbidity Score^b^ (*n* (%))				
0	2806 (62.7)	6112 (72.0)	14 950 (64.7)	33 052 (71.8)
1	764 (17.1)	1167 (13.8)	3666 (15.9)	6168 (13.4)
2	484 (10.8)	756 (8.9)	2596 (11.2)	4342 (9.4)
⩾3	422 (9.4)	453 (5.4)	1908 (8.3)	2484 (5.4)

*n,*  number of persons.

a Income,Total disposable personal income in the year prior to inclusion. Deciles are calculated across the total study population per calendar year.

b Cumulated Charlson Comorbidity Score up to 5 years prior to inclusion.

### Person-years at risk

The participants contributed with a total of 642 914.8 PY. Of these, widowed persons contributed with 223 715.3 PY (of which women contributed with 78.8%) and married persons contributed with 419 199.5 PY (of which women contributed with 65.7%). Each person contributed only with PY according to their current marital status at all times. A total of 26 143 (37.8%) of those who were matched as married (see Table 1) changed exposure status during the study. Of these, 4652 were men (20.1% of all married men), and 21 491 were women (46.7% of all married women). The mean age was 85.1 (s.d. 6.2) and 83.1 (s.d. 6.0) years for men and women, respectively, at the time of re-inclusion into the study as widowed.

### Overall mortality

The total number of deaths was 42 962. Of these, 55.0% (*n* = 23 620) were married and 45.0% (*n* = 19 342) were widowed at the time of death. The overall MRR [95% CI] for widowed persons compared to married persons was 1.25 [1.23–1.28] (*p* < 0.001), adjusted for sex, age, income, CCS and calendar year. For men, the adjusted MRR was 1.29 [1.24–1.33] (*p* < 0.001) and for women it was 1.23 [1.20–1.26] (*p* < 0.001).

#### Mortality by age strata

Table 2 shows the MRs and MRRs stratified according to age and sex. For both men and women, MRRs appeared to decrease with increasing age. Mortality was higher in widowed men compared to married men in all age strata until ⩾95 years where there was no longer a significant difference. For women, mortality was higher in the widowed compared to the married until ⩾90 years of age. For women ⩾100 years of age, widowed women had a significantly lower mortality compared to married women (MRR 0.31 [0.15–0.65], *p* < 0.001).

**Table 2.** Mortality rates per 10 000 person-years and mortality rate ratios of widowed persons compared to married persons stratified according to 5-year age strata and sex

	Widowed		Married		MRR [95% CI]^a^	
Age	Male	Female	Male	Female	Male	Female
65–69, *n* deaths	76	131	211	349	2.26 [1.72–2.97]	1.98 [1.61–2.43]
PY	2194.3	6675.9	10 589.5	27 045.4		
MR	346.4	196.2	199.3	129.0		
70–74, *n* deaths	278	614	835	1286	1.76 [1.53–2.03]	1.64 [1.48–1.81]
PY	6415.6	23 043.9	27 294.1	67 195.3		
MR	433.3	266.5	305.9	191.4		
75–79, *n* deaths	720	1663	1810	2451	1.71 [1.56–1.87]	1.46 [1.37–1.55]
PY	10 284.6	41 323.9	37 553.4	80 645.5		
MR	700.1	402.4	482.0	303.9		
80–84, *n* deaths	1287	3075	3046	3327	1.40 [1.31–1.50]	1.26 [1.20–1.33]
PY	12 001.4	48 154.5	35 973.1	61 972.6		
MR	1072.4	638.6	846.7	536.9		
85–89, *n* deaths	1697	4157	3346	2948	1.18 [1.11–1.25]	1.18 [1.12–1.24]
PY	10 190.6	38 175.1	22 920.9	30 395.4		
MR	1665.3	1088.9	1459.8	969.9		
90–94, *n* deaths	1306	2879	1932	1323	1.13 [1.05–1.21]	1.05 [0.98–1.13]
PY	5036.8	15 989.8	8092	7414.6		
MR	2592.9	1800.5	2387.5	1784.3		
95–99, *n* deaths	458	876	451	255	1.11 [0.97–1.27]	0.99 [0.85–1.15]
PY	1174.3	2801.2	1259.6	764.7		
MR	3900.2	3127.2	3580.5	3334.6		
⩾100, *n* deaths	55	70	31	19	0.74 [0.44–1.25]	0.31 [0.15–0.65]
PY	110.5	143.0	54.0	29.5		
MR	4977.4	4895.1	5740.7	6440.7		

PY, person-years at risk; *n*, number of deaths; MR, mortality rate pr. 10 000 PY.

MRR, mortality rate ratio for widowed compared to married; CI, confidence interval.

a Adjusted for calendar year, income, Charlson Comorbidity Score.

#### Mortality by time since spousal loss

**Fig. 1.**
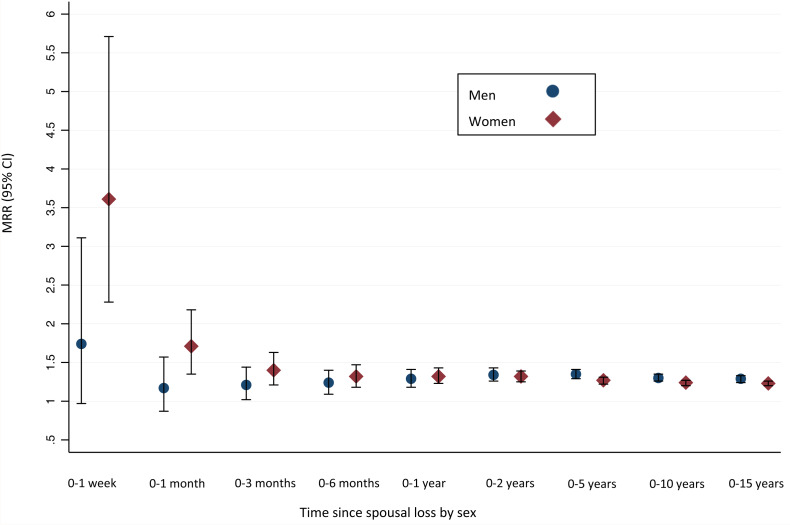
Mortality rate ratios for widowed persons compared to married persons by sex and time since spousal loss. MRR, mortality rate ratio adjusted for age, calendar year, Charlson Comorbidity Score and income. 95% CI, 95% confidence interval.

Table 3 and Fig. 1 show the sex-specific MRs and MRRs by time since spousal loss. For women, the MRR was highest in the first week (MRR 3.61 [2.30–5.66], *p* < 0.001). For men, there was no significant difference in mortality between the widowed and married group in the follow-up periods including only the first week or month, respectively. After a follow-up period of up to 3 months, mortality was increased for the widowed men (MRR 1.21 [1.02–1.44], *p* = 0.03), and this remained so for all the subsequent durations of follow-up with similar MRRs and CIs.

**Table 3.** Mortality rates per 10 000 person-years and mortality rate ratios stratified according to marital status, sex and time since spousal loss

	Widowed		Married		MRR [95% CI]^a^	
	Male	Female	Male	Female	Male	Female
0–1 week, *n* (deaths)	28	83	33	30	1.74 [1.00**–**3.02]	3.61 [2.30**–**5.66]
PY	200	657.2	507.5	1010.2		
MR	1400	1262.9	650.2	297.0		
0–1 month, *n* (deaths)	93	216	158	148	1.17 [0.88**–**1.56]	1.71 [1.35**–**2.71]
PY	782.5	2572.6	1986.4	3956.2		
MR	1188.5	839.6	795.4	374.1		
0–3 months, *n* (deaths)	257	536	437	435	1.21 [1.02**–**1.44]	1.40 [1.21**–**1.62]
PY	2271.7	7488	5779	11 503.4		
MR	1131.3	715.8	756.2	378.1		
0–6 months, *n* (deaths)	496	1018	837	881	1.24 [1.10**–**1.40]	1.32 [1.19**–**1.46]
PY	4444.4	14 710.8	11 342	22 558.2		
MR	1116.0	692.0	738.0	390.5		
0–1 year, *n* (deaths)	981	1958	1607	1717	1.29 [1.18**–**1.41]	1.32 [1.23**–**1.42]
PY	8573.7	28 609.8	22 058.4	43 774.1		
MR	1144.2	684.4	728.5	392.2		
0–2 years, *n* (deaths)	1857	3760	3110	3276	1.34 [1.26**–**1.43]	1.32 [1.25**–**1.39]
PY	15 966.8	54 204.6	41 876.1	82 792.7		
MR	1163.0	693.7	742.7	395.7		
0–5 years, *n* (deaths)	3919	8066	6880	7124	1.35 [1.29**–**1.41]	1.27 [1.22**–**1.31]
PY	32 175.8	114 416	89 786.8	175 939.2		
MR	1218.0	705.0	766.3	404.9		
0–10 years, *n* (deaths)	5504	12 328	10 590	10 952	1.30 [1.26**–**1.35]	1.24 [1.20**–**1.27]
PY	44 747	165 054.5	133 463.3	257 913.4		
MR	1230.0	746.9	793.5	424.6		
0–15 years, *n* (deaths)	5871	13 449	11 645	11 941	1.29 [1.24**–**1.33]	1.23 [1.20**–**1.26]
PY	47 368.5	176 139.9	143 554.2	275 210.3		
MR	1239.4	763.5	811.2	433.9		
Total follow-up, 16 years, *n* (deaths)	5877	13 465	11 662	11 958	1.29 [1.24–1.33]	1.23 [1.20–1.26]
PY	47 408.1	176 307.3	143 736.6	275 462.9		
MR	1239.7	763.7	811.3	434.1		

PY, person years at risk; *n*, number of deaths; MR, mortality rate pr. 10 000 PY.

MRR, mortality rate ratio for widowed compared to married; CI, confidence interval.

a Adjusted for calendar year, age, income, Charlson Comorbidity Score.

### Cause-specific mortality

**Fig. 2.**
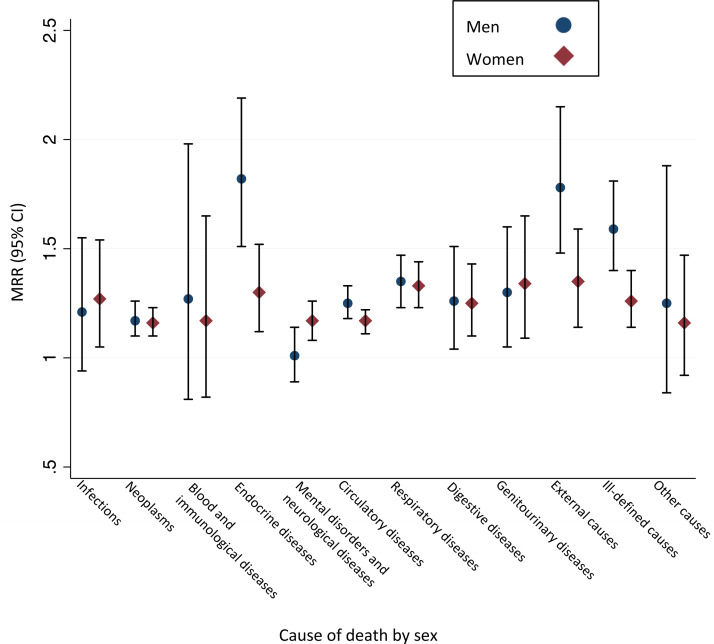
Cause-specific mortality rate ratios for widowed persons compared to married persons stratified according to sex. MRR, mortality rate ratio adjusted for age, calendar year, Charlson Comorbidity Score and income. 95% CI, 95% confidence interval.

Table 4 and Fig. 2 show the sex-specific MRs and MRRs for specific causes of death. For women, widowhood was associated with increased cause-specific mortality for each of the considered causes of death, except for death from diseases of the blood or immunological system, and from death from other causes. For men, there was no difference in cause-specific mortality for death from infections, diseases of the blood or immunological system, from neurological diseases or mental disorders or from the category other diseases. For all remaining causes of death, mortality was significantly increased in the widowed men compared to the married men.

**Table 4.** Mortality rates for specific causes of death per 10 000 person-years stratified according to marital status and sex. Cause-specific mortality rate ratios for widowed persons compared to married persons.

	Widowed		Married		MRR [95% CI]^a^		
	Male	Female	Male	Female	Both sexes	Male	Female
PY^b^	47 364.8	176 247.5	143 597.2	275 286.4			
Cause of death							
Infections, *n* (deaths)	104	268	185	207	1.25 [1.07–1.45]	1.21 [0.94–1.55]	1.27 [1.05–1.54]
MR	22.0	15.2	12.9	7.5			
Neoplasms, *n* (deaths)	1226	2603	2971	3111	1.16 [1.11–1.21]	1.17 [1.10–1.26]	1.16 [1.10–1.23]
MR	258.8	147.7	206.9	113.0			
Blood and immunological diseases, *n* (deaths)	34	84	53	62	1.21 [0.92–1.59]	1.27 [0.81–1.98]	1.17 [0.82–1.65]
MR	7.2	4.8	3.7	2.3			
Endocrine diseases, *n* (deaths)	203	418	303	339	1.51 [1.34–1.70]	1.82 [1.51–2.19]	1.30 [1.12–1.52]
MR	42.9	23.7	21.1	12.3			
Neurological diseases or mental disorders, *n* (deaths)	423	1685	904	1245	1.13 [1.05–1.20]	1.01 [0.89–1.14]	1.17 [1.08–1.26]
MR	89.3	95.6	63.0	45.2			
Circulatory diseases, *n* (deaths)	1954	4390	3855	3623	1.21 [1.17–1.26]	1.25 [1.18–1.33]	1.17 [1.11–1.22]
MR	412.5	249.1	268.5	131.6			
Respiratory diseases, *n* (deaths)	793	1517	1481	1303	1.33 [1.26–1.41]	1.35 [1.23–1.47]	1.33 [1.23–1.44]
MR	167.4	86.1	103.1	47.3			
Digestive diseases, *n* (deaths)	185	531	363	448	1.26 [1.13–1.40]	1.26 [1.04–1.51]	1.25 [1.10–1.43]
MR	39.1	30.1	25.3	16.3			
Genitourinary diseases, *n* (deaths)	155	234	254	165	1.31 [1.13–1.52]	1.30 [1.05–1.60]	1.34 [1.09–1.65]
MR	32.7	13.3	17.7	6.0			
External causes, *n* (deaths)	211	383	277	262	1.53 [1.35–1.74]	1.78 [1.48–2.15]	1.35 [1.14–1.59]
MR	44.5	21.7	19.3	9.5			
Ill-defined causes, *n* (deaths)	441	1013	585	640	1.40 [1.29–1.52]	1.59 [1.40–1.81]	1.26 [1.14–1.40]
MR	93.1	57.5	40.7	23.2			
Other causes, *n* (deaths)	38	179	77	148	1.19 [0.97–1.45]	1.25 [0.84–1.88]	1.16 [0.92–1.47]
MR	8.0	10.2	5.4	5.4			

PY, person-years at risk; n, number of deaths; MR, mortality rate pr. 10 000 PY.

MRR, mortality rate ratio for widowed compared to married; CI, confidence interval.

a Adjusted for age, income and Charlson Comorbidity Score. MRRs for both sexes are also adjusted for sex.

b A total of 1029 causes of death were missing. These observations are excluded from the cause of death analysis and the PY presented in this table.

## Discussion

We examined the association between widowhood and mortality in a Danish study population aged 65 years and above for up to 16 years of follow-up. The increase in the overall MR in widowed persons was 25% (23% in women and 29% in men). This is similar to what has been found in a meta-analysis by Shor *et al*. (2012), but higher than the 11% increased risk found in the meta-analyses by Moon *et al*. (2011) and Manzoli (2007).

We found a significant widowhood effect on mortality until age ⩾90 years for women and ⩾95 years for men. The widowhood effect appeared to decrease with increasing age. Shor *et al*. (2012) found results similar to ours with decreasing MRR with increasing age. They divided age into 10-year age intervals up to ⩾80 years of age and found significant differences in mortality between widowed and married in all age groups (Shor *et al*., 2012). They predicted that by ⩾90 years, the difference would be insignificant (Shor *et al*., 2012). Currently, almost 20% of the Danish population is aged 65 years or above, and about 4.6% is aged 80 years or above (Statistics Denmark, 2020). Statistics Denmark has predicted that in 2050, these numbers will be about 25 and 9.5%, respectively (Statistics Denmark, 2020). Thus, it is especially the proportion of the oldest old which is increasing. This overlap between most widowed people being of older age (The Loomba Foundation, 2015) and the fact that the proportion of those of older age is increasing in most societies (United Nations, 2017) underlines the importance of this finding.

We found that for women ⩾100 years of age, widowhood was associated with a lower risk of mortality. Although the number of PY is relatively low in this age stratum (337 PY in total), the phenomenon is interesting and has not previously been shown. Marriage is known to be protective of health in general (Kiecolt-Glaser and Newton, 2011); however, the protective effect of marriage on health diminishes with decreasing health (Zheng and Thomas, 2013). Further, a study has found that widowed women have less risk of becoming frail than married women in a follow-up period of 3 years (Wilcox *et al*., 2003). As health often decreases with increasing age, it is possible that in very old age, the protective effect of marriage on women compared to widowed women is no longer present. Indeed, marriage might even be a health disadvantage, because married women might become caregivers for an ill spouse, which is known to be associated with poor health outcomes (Penning and Wu, 2016; de Zwart *et al*., 2017). Another reason could be that widowed women who live to be 100 years, despite becoming widowed, belong to a particularly resilient group.

Shor *et al*. (2012) concluded in their meta-analysis that the association between widowhood and mortality was strongest during the first 2 years of widowhood, after which it decreased but remained significant for up to 20 years. We did not find a similar temporal association in the present study, where we were able to examine different lengths of widowhood in the same study population. For women, the MRR was highest during the first month of widowhood. When including up to 3 months, the MRR did no longer appear to be different from the one including up to 15 years of widowhood. This indicates that the overall increased mortality which is seen in widowed women compared to married women may be explained by an increased mortality within the first months of widowhood. In men, the MRR was insignificant during the first month. The non-significance did not seem to be explained by a lack of statistical power. A possible explanation is the high age of the people included in the present study compared to previous studies. Adjacic-Gross *et al*. (2008) examined the risk of suicide in widowed people compared to married people and found that the risk was highest in the first week of widowhood, after which it decreased but remained high in the first year. The increase in mortality was higher in the younger participants than in those of older age – especially in the first week of widowhood (Adjacic-Gross *et al*., 2008). A similar association was found by Brenn and Ytterstad (2016), who also found a strong association of death from external causes immediately after spousal death, which was highest in the youngest age group examined and seemed to decrease with increasing age. The total number of deaths from external causes is relatively low compared to deaths from other causes both in the present study and in the study by Brenn and Ytterstad (2016). Nonetheless, the relative increase in the risk of mortality in widowed people compared to married people from this cause is high. In the study by Adjacic-Gross *et al*. (2008), the SMR was about 15 for widowed men ⩾60 years of age the first week and almost 90 for those under 60 years of age (Adjacic-Gross *et al*., 2008). Similarly, the HR for widowed men *v*. married men aged 55–64 years was above 15 for death from external causes in the first week after widowhood in the study by Brenn and Ytterstad (2016). Thus, although the total number of deaths from this cause is low, its relative contribution to the increased risk of mortality in early widowhood is high. In comparison, we found a lower MRR from death from external causes for widowed men compared to married men which was about 1.8. Thus, it might be that the increased risk of mortality immediately after spousal loss previously seen in widowed men is explained primarily by the very high risk of death from external causes immediately after spousal loss in younger samples. Nonetheless, despite this difference, external causes are still an important cause of death in widowed people also at older age. Since suicide most often occurs in people who are depressed (Hawton and van Heeringen, 2009), and the prevalence of depression in widowed people is known to be high (Kristiansen *et al*., 2019*a*, 2019b), a possible target for preventive interventions in terms of mortality in widowhood could be depression and consequently suicide in widowed people.

In line with previous research, we found that mortality was increased in widowed persons for most specific causes of death (Elwert and Christakis, 2008; Brenn and Ytterstad, 2016). Elwert and Christakis (2008) examined a large number of specific causes of death and found that widowhood increased mortality by both acute events such as sepsis, pneumonia and influenza as well as chronic diseases such as congestive heart failure, diabetes and chronic obstructive pulmonary disease. The increased MR in widowed persons across most causes of death indicates that widowhood may be considered a general risk to the health. It has been shown that some physical health disparities may occur prior to widowhood (Wilcox *et al*., 2003; Einio *et al*., 2017). In the present study, we controlled for comorbidity 5 years before inclusion using register-based data with very high coverage of all hospital contacts (Schmidt *et al*., 2015). The distribution of CCS at time of matching appeared to be similar in widowed and married persons (see Table 1). Thus, if the increased mortality in widowed persons was explained by health disparities prior to widowhood, these are not evident from the hospital records (in this study represented by data from the National Patient Registers used in the CCS), indicating that the diseases were either diagnosed and treated in primary care (as information from primary care is not included in the National Patient Register) or not diagnosed and treated at all in widowed persons. The latter might be important as qualitative research has suggested that widowed people who have been caregivers of an ill spouse may deliberately have postponed taking care of their own health problems (DiGiacomo *et al*., 2013). It could also be that the differences in health disparities prior to widowhood which have previously been shown (Wilcox *et al*., 2003; Einio *et al*., 2017) are less pronounced in a Danish sample, as all people in Denmark have the same universal and free access to the healthcare system, reducing differences in health due to for an example SES. The study by Einio *et al*. is however conducted in Finland, and as the two countries are comparable regarding both healthcare (Eide *et al*., 2017), as well as the association of income and mortality in older age (Mortensen *et al*., 2016), this does not seem to be the full explanation. In order to further explore the influence of the time before spousal death, future studies should include information on the health, as well as the causes of death from both spouses. This could enlighten possible effects from caregiving, as well as effects of specific causes of deaths from the predeceased spouse such as expected *v*. unexpected deaths, all factors which have been proposed to explain some of the widowhood effect (Elwert and Christakis, 2008; Sullivan and Fenelon, 2014; Siflinger, 2017).

Cause-specific mortality from the ICD-10 chapter ‘symptoms, signs and ill-defined causes’ was increased in widowed persons for both sexes. There are no previous studies examining death from unknown and ill-defined causes in widowed persons. This might be because the World Health Organization discourage the use of the ICD-10 codes R00–R99 (symptoms, signs and ill-defined causes) as the cause of death unless all measures have been taken to establish the cause of death (Ylijoki-Sorensen *et al*., 2014). The occurrence of the R00–R99 codes in death certificates in Denmark might be due to a low rate of autopsies (Helweg-Larsen, 2011). A study found that people over 70 years were the most likely age group to be registered with an unknown or ill-defined cause of death and least likely to be autopsied (Ylijoki-Sorensen *et al*., 2014). The authors speculated that doctors might not have been as meticulous about finding the correct cause of death in older people (Ylijoki-Sorensen *et al*., 2014).

### Limitations

The study has some limitations. First, since the persons who were reincluded as widowed must be of older age at the time of widowhood than they were at the time of matching, the mean age in widowed persons in the analyses is higher than in married persons. To account for this bias, all analyses were either stratified according to or adjusted for age. However, since age becomes an increasingly dominating risk factor of mortality as age increases, some age-related residual confounding in the age-adjusted analyses cannot be ruled out.

Another limitation is the use of personal yearly income as the only measure of SES. Other measures of SES, including wealth and education, have been found to account for up to a third of the increased mortality in widowed people in an American study (Sullivan and Fenelon, 2014). A study from the Netherlands did not find that income was associated with mortality in widowed people (van den Berg *et al*., 2011). Considering the extensive welfare system in Denmark, the influence of income in widowhood might be similar to that of the Netherlands. Thus, using income alone as a proxy for SES might not be sufficient and there might be residual confounding due to other measures of SES. However, due to the age range of the study population, it was not possible to meaningfully adjust for educational level or latest employment status. The educational attainment register was not fully operating until the 1970s; therefore, many persons in the study population had finished their education before the register had sufficient coverage (Statistics Denmark, 2019). Similarly, due to the age of the study population, the recent employment status was not meaningful, as the majority of the study population was retired.

The study aimed to examine mortality in widowed people at older age specifically compared to married people, as losing a spouse to death is a frequent event in older age. The results of the study are therefore restricted to enlightening the relationship between mortality in widowed and married people only. Other groups of people could have been considered for comparison including singletons, people cohabiting with another person than a spouse, as well as a comparison of those who stay widowed and those who remarry. For future studies, including other exposure groups besides married and widowed people could be relevant in order to further understand the causality of the increased mortality in widowed people compared to married people.

## Conclusion

We found an increased mortality in widowed persons compared to married persons and have shown that the increased mortality persists until very old age, however, is no longer significant at ages over 90 and 95 years for women and men, respectively. Mortality is increased immediately after widowhood for women, whereas for men the effect of widowhood on mortality seems less dependent on the time since spousal loss. Widowhood was associated with increased mortality from most specific causes of death, with the largest effect on death from endocrine diseases and death from external causes, including suicides and accidents.

## Data Availability

Data have been made available for the first author specifically via Statistics Denmark and can therefore not be shared.

## Appendix

Project protocol: Widowhood-associated mortality

### Background

Increased mortality associated with widowhood, also known as the ‘widowhood effect’, is a well-known phenomenon, documented most recently in two meta-analyses (Moon *et al.*, 2011, Shor *et al.*, 2012). Many studies upon the widowhood effect have been conducted (Moon et al., 2011, Shor et al., 2012); however, the comparability of these studies is often limited due to differences in the methods, samples and adjustment for possible mediators and confounders. Many factors might influence the outcome of widowhood both as risk factors and protective factors. In order to understand this phenomenon properly, these factors need to be investigated.

### Aims of the study

The aim of this study is to examine the risk of mortality associated with widowhood both regarding all-cause and specific causes of death. Furthermore, to examine the effect of age and gender on the mortality risk. Lastly, to examine if there is an association between time since spousal death and mortality risk.

### Methods

#### Design

A register-based population-based cohort study. The exposure studied is becoming widowed.

#### Population

All people in Denmark who are married and became widowed at the age of 65 years and above in the period 2000–2009.

#### Sample

A cohort consisting of a 10% random sample of those who were widowed at age 65 years and above in 2000–2009 is created.

For each widowed person, five persons who match the widowed on age (±2 years) and gender, and who are married at the time the widowed person lost his/her spouse are included by random selection.

#### Study period

The exposed are included from the date they become widowed and are followed up until death (outcome), or end of study on December 31 2015. If a participant remarries, he/she is censored.

The unexposed are included from the day the exposed became widowed, and are followed up either until death (outcome) or end of study on 31 December 2015. If a non-exposed becomes widowed, he/she is censored from the study, as he/she is no longer unexposed.

#### Material

By cross-linkage of different registers (via the CPR-number), data from the following registers are combined: The Danish Civil Registration System (CPR-register), The Danish Register of Causes of Death, The National Patient Register (Landspatientregisret), The Danish Income Register, The Danish Educational Attainment Register. For measures used in the study, please see below.

#### Measures

##### Exposure status

The exposure examined in this study is losing a spouse to death, that is, becoming widowed. Exposure status is assessed from the Danish Civil Registration System, where a yearly update of marital status is registered. People who lose their spouse to death are included as exposed, and for each exposed person, five people who are still married at the time are included as non-exposed.

##### Outcome

The outcome examined in this study is death. Time and cause of death are assessed from the Danish Civil Registration System. Time of death is registered at date-level. Cause of death is registered according to ICD-10. For this study, the causes of death are grouped according to ICD-10 domains: (1) Infections and parasitic diseases [A00–B99], (2) Neoplasms [C00–D48], (3) Diseases of the blood and blood-forming organs and immunological disorders [D50–D89], (4) Endocrine, nutritional and metabolic diseases [E00–E90], (5) Mental and behavioural disorders [F00–F99], (6) Diseases of the nervous system and sense organs [G00–H95], (7) Diseases of the circulatory system [I00–I99], (8) Diseases of the respiratory system [J00–J99], (9) Diseases of the digestive system [K00–K93], (10) Diseases of the skin and subcutaneous tissue [L00–L99], (11) Diseases of the musculoskeletal and connective tissue [M00–M99], (12) Diseases of the genitourinary system [N00–N99], (13) Congenital malformations and chromosomal abnormalities [Q00–Q99], (14) Symptoms signs and ill-defined causes [R00–R99], (15) Injury, poisoning and other external causes of morbidity and mortality [S00–Y98].

##### Covariates

The following covariates are used in the study: age, gender, socioeconomic status (SES), physical comorbidity. Age and gender are assessed in the Danish Civil Registration System.

*SES:* Is defined from total personal yearly income from the Danish Income Register and from highest education attained from the Danish Educational Attainment Register. Income is registered in DKKR.

The highest education attained is in accordance with the International Standard Classification of Education (ISCED 2011), and is divided into the following five groups: level 1 + 2: primary school, level 3: secondary school, level 5: short-cycle tertiary education, level 6: bachelor or equivalent, level 7 + 8: master or equivalent, or above. ISCED 2011 level 4 has no common Danish equivalent and is not used in the register.

*Comorbidity:* Data on physical comorbidity is obtained from the National Patient Register. Diagnoses are registered according to ICD-10. Comorbidity is calculated according to an adapted version of the Charlson Comorbidity Index (see Table A1). Diagnoses given up to 5 years prior to inclusion are cumulated into the Charlson score for each participant.

**Table A1.** Charlson Comorbidity Index

Comorbidity	Score	ICD codes
Myocardial infarct	1	I21–I23
Congestive heart failure	1	I11, I13.0, I13.2, I50
Peripheral vascular disease	1	I70–I74, I77
Cerebrovascular disease	1	I60–I69, G45, G46
Dementia	1	F00–F03, G30
Chronic pulmonary disease	1	J40–J47, J60–J67, J68.4, J70.1, J70.3, J84.1, J92.0, J96.1, J98.2, J98.3,
Connective tissue disease	1	M05, M06, M08, M09, M30–M36, D86
Ulcer disease	1	K22.1, K25–K28
Mild liver disease	1	B18, K70.0–K70.3, K70.9, K71, K73, K74, K76.0
Diabetes without end organ disease	1	E10.0, E10.1, E10.9, E11.0, E11.1, E11.9, E14.0, E14.1, E14.9
Hemiplegia and paraplegia	2	G81, G82
Moderate or severe renal disease	2	I12, I13, N00–N05, N07, N11, N14, N17–N19, Q61
Diabetes with end-organ disease	2	E10.2–E10.8, E11.2–E11.8, E14.2–E14.8
Any tumour	2	C00–C76
Leukaemia	2	C91–C95, D45
Lymphoma	2	C81–C85, C88, C90, C96
Moderate or severe liver disease	3	B15.0, B16.0, B16.2, B19.0, K70.4, K72, K76.6, I85
Metastatic solid tumour	6	C77–C80
AIDS	6	B21, B22, B23, B24

#### Statistical analyses

##### Mortality

*Overall mortality:* Mortality rates will be calculated for both the exposed and non-exposed. The rates will be presented both as total and gender stratified with 95% confidence intervals. Five-year age and gender-stratified mortality rates will be calculated for both the exposed and non-exposed. Cox regression models will be used to adjust for confounders.

*Specific cause of death:* Mortality rates for each specific cause of death according to the groups specified above will be calculated for both the exposed and non-exposed.

Both the overall mortality and the rates for specific causes of death will be adjusted for age, gender, SES and comorbidity. The age and gender-stratified mortality rates will be adjusted for SES and comorbidity.

*Time since bereavement:* Mortality rates as a function of time since bereavement (=time since inclusion) will be calculated. A mortality rate ratio between the exposed and non-exposed will be calculated and a curve with 95% confidence intervals will be presented.

##### Planned publication

‘Widowhood associated cause-specific mortality in older age’,

Authors: Christina Blanner Kristiansen, Anna Mejldal, Matthew Prina, Povl Munk-Jørgensen, Annette Kjær Ersbøll, Kjeld Andersen.

## References

[ref1] Ajdacic-Gross V, Ring M, Gadola E, Lauber C, Bopp M, Gutzwiller F and Rössler W (2008) Suicide after bereavement: an overlooked problem. Psychological Medicine 38, 673–676. 18226288 10.1017/S0033291708002754

[ref2] Baadsgaard M and Quitzau J (2011) Danish registers on personal income and transfer payments. Scandinavian Journal of Public Health 39, 103–105. 21775365 10.1177/1403494811405098

[ref3] Brenn T and Ytterstad E (2016) Increased risk of death immediately after losing a spouse: cause-specific mortality following widowhood in Norway. Preventive Medicine 89, 251–256. 27311340 10.1016/j.ypmed.2016.06.019

[ref4] Brink M, Green A, Bojesen AB, Lamberti JS, Conwell Y and Andersen K (2017) Physical health, medication, and healthcare utilization among 70-year-old people with schizophrenia: a nationwide Danish register study. American Journal of Geriatric Psychiatry 25, 500–509. 28215901 10.1016/j.jagp.2016.12.015

[ref5] Charlson ME (1987) A new method of classifying prognostic comorbidity in longitudinal studies: development and validation. Journal of Chronic Diseases 40, 373–383. 3558716 10.1016/0021-9681(87)90171-8

[ref6] Charlson M, Szatrowski TP, Peterson J and Gold J (1994) Validation of a combined comorbidity index. Journal of Clinical Epidemiology 47, 1245–1251. 7722560 10.1016/0895-4356(94)90129-5

[ref7] de Zwart PL, Bakx P and van Doorslaer EKA (2017) Will you still need me, will you still feed me when I'm 64? The health impact of caregiving to one's spouse. Health Economics 26, 127–138. 28940916 10.1002/hec.3542 PMC5639350

[ref8] DiGiacomo M, Lewis J, Nolan MT, Phillips J and Davidson PM (2013) Transitioning from caregiving to widowhood. Journal of Pain and Symptom Management 46, 817–825. 23571208 10.1016/j.jpainsymman.2013.01.005

[ref9] Eide TB, Straand J, Björkelund C, Kosunen E, Thorgeirsson O, Vedsted P and Rosvold EO (2017) Differences in medical services in Nordic general practice: a comparative survey from the QUALICOPC study. Scandinavian Journal of Primary Health Care 35, 153–161. 28613127 10.1080/02813432.2017.1333323 PMC5499315

[ref10] Einio E and Martikainen P (2019) Risk of hospitalization for cancer, musculoskeletal disorders, injuries, or poisonings surrounding widowhood. American Journal of Epidemiology 188, 110–118. 30137200 10.1093/aje/kwy184

[ref11] Einio E, Moustgaard H, Martikainen P and Leinonen T (2017) Does the risk of hospitalisation for ischaemic heart disease rise already before widowhood? Journal of Epidemiology and Community Health 71, 599–605. 28235819 10.1136/jech-2016-207987

[ref12] Elwert F and Christakis NA (2008) The effect of widowhood on mortality by the causes of death of both spouses. American Journal of Public Health 98, 2092–2098. 18511733 10.2105/AJPH.2007.114348 PMC2636447

[ref13] Ennis J and Majid U (2019) ‘Death from a broken heart’: a systematic review of the relationship between spousal bereavement and physical and physiological health outcomes. Death Studies, 1–14. 10.1080/07481187.2019.1661884 31535594

[ref14] Hawton K and van Heeringen K (2009) Suicide. The Lancet 373, 1372–1381. 10.1016/S0140-6736(09)60372-X 19376453

[ref15] Helweg-Larsen K (2011) The Danish register of causes of death. Scandinavian Journal of Public Health 39, 26–29. 10.1177/1403494811399958 21775346

[ref16] Kiecolt-Glaser JK and Newton TL (2011) Marriage and health: his and hers. Psychological Bulletin 127, 472–503. 10.1037/0033-2909.127.4.472 11439708

[ref17] Kristiansen CB, Kjær JN, Hjorth P, Andersen K and Prina AM (2019 *a*) Prevalence of common mental disorders in widowhood: a systematic review and meta-analysis. Journal of Affective Disorders 245, 1016–1023. 30699843 10.1016/j.jad.2018.11.088

[ref18] Kristiansen CB, Kjær JN, Hjorth P, Andersen K and Prina AM (2019 *b*) The association of time since spousal loss and depression in widowhood: a systematic review and meta-analysis. Social Psychiatry and Psychiatric Epidemiology 54, 781–792. 30887075 10.1007/s00127-019-01680-3

[ref19] Manzoli LL (2007) Marital status and mortality in the elderly: a systematic review and meta-analysis. Social Sciences and Medicine 64, 77–94. 10.1016/j.socscimed.2006.08.031 17011690

[ref20] Moon JR, Kondo N, Glymour MM and Subramanian SV (2011) Widowhood and mortality: a meta-analysis. PLoS ONE 6, e23465. 21858130 10.1371/journal.pone.0023465 PMC3157386

[ref21] Mortensen LH, Rehnberg J, Dahl E, Diderichsen F, Elstad JI, Martikainen P, Rehkopf D, Tarkiainen L and Fritzell J (2016) Shape of the association between income and mortality: a cohort study of Denmark, Finland, Norway and Sweden in 1995 and 2003. BMJ Open 6, e010974. 10.1136/bmjopen-2015-010974 PMC5223725 28011804

[ref22] National Committee on Health Research Ethics (2019) What to notify?. Available at http://en.nvk.dk/how-to-notify/what-to-notify (Accessed 10 December 2019).

[ref23] Pedersen CB (2006) The Danish Civil Registration System. A cohort of eight million persons. Danish Medical Bulletin 53, 441–449. 17150149

[ref24] Penning MJ and Wu Z (2016) Caregiver stress and mental health: impact of caregiving relationship and gender. The Gerontologist 56, 1102–1113. 26035875 10.1093/geront/gnv038

[ref25] Schmidt M, Schmidt SAJ, Sandegaard JL, Ehrenstein V, Pedersen L and Sørensen HT (2015) The Danish National Patient Registry: a review of content, data quality, and research potential. Clinical Epidemiology 7, 449–490. 26604824 10.2147/CLEP.S91125 PMC4655913

[ref26] Shor E, Roelfs DJ, Curreli M, Clemow L, Burg MM and Schwartz JE (2012) Widowhood and mortality: a meta-analysis and meta-regression. Demography 49, 575–606. 22427278 10.1007/s13524-012-0096-x PMC3640496

[ref27] Siflinger B (2017) The effect of widowhood on mental health – an analysis of anticipation patterns surrounding the death of a spouse. Health Economics 26, 1505–1523. 27747997 10.1002/hec.3443

[ref28] Stata Statistical Software (2017) Release 15. College Station, TX: StataCorp LP.

[ref29] Statistics Denmark (2019) Highest education attained. Available at https://www.dst.dk/en/Statistik/dokumentation/documentationofstatistics/highest-education-attained (Accessed 10 December 2019).

[ref30] Statistics Denmark (2020) Statbank.dk (Accessed 20 May 2020).

[ref31] Sullivan AR and Fenelon A (2014) Patterns of widowhood mortality. The Journals of Gerontology Series B: Psychological Sciences and Social Sciences 69, 53–62. 10.1093/geronb/gbt079 PMC3968855 24077660

[ref32] The Loomba Foundation (2015) The Global Widows Report 2015. Available at https://www.theloombafoundation.org/sites/default/files/2019-06/WWR.pdf (Accessed 10 December 2019).

[ref33] United Nations (2017) Department of Economic and Social Affairs, Population Division. World Population Prospects: The 2017 Revision, Key Findings and Advance Tables. ESA/P/WP/248.

[ref34] Vable AM, Subramanian SV, Rist PM and Glymor MM (2015) Does ‘the widowhood effect’ precede spousal bereavement? Results from a nationally representative sample of older adults. The American Journal of Geriatric Psychiatry 23, 283–292. 24974142 10.1016/j.jagp.2014.05.004 PMC5511695

[ref35] van den Berg GJ, Lindeboom M and Portrait F (2011) Conjugal bereavement effects on health and mortality at advanced ages. Journal of Health Economics 30, 774–794. 21715034 10.1016/j.jhealeco.2011.05.011

[ref36] Wilcox S, Evenson KR, Aragaki A, Wassertheil-Smoller S, Mouton CP and Loevinger BL (2003) The effects of widowhood on physical and mental health, health behaviors, and health outcomes: The Women's Health Initiative. Journal of Health Psychology 22, 513–522. 10.1037/0278-6133.22.5.513 14570535

[ref37] World Health Organization (1992) The ICD-10 Classification of Mental and Behavioural Disorders: Clinical Descriptions and Diagnostic Guidelines. Geneva: World Health Organization.

[ref38] Ylijoki-Sorensen S, Sajantila A, Lalu K, Bøggild H, Boldsen JL and Boel LW (2014) Coding ill-defined and unknown cause of death is 13 times more frequent in Denmark than in Finland. Forensic Science International 244, 289–294. 25300069 10.1016/j.forsciint.2014.09.016

[ref39] Zheng H and Thomas PA (2013) Marital status, self-rated health, and mortality: overestimation of health or diminishing protection of marriage? Journal of Health and Social Behavior 54, 128–143. 23321283 10.1177/0022146512470564 PMC9052865

